# Reliability and validity of triage systems in paediatric emergency care

**DOI:** 10.1186/1757-7241-17-38

**Published:** 2009-08-27

**Authors:** Mirjam van Veen, Henriette A Moll

**Affiliations:** 1 Department of Paediatrics, Erasmus MC-Sophia Children's Hospital, University Medical Center Rotterdam, PO Box 2060, 3000 CB Rotterdam, The Netherlands

## Abstract

**Background:**

Triage in paediatric emergency care is an important tool to prioritize seriously ill children. Triage can also be used to identify patients who do not need urgent care and who can safely wait. The aim of this review was to provide an overview of the literature on reliability and validity of current triage systems in paediatric emergency care

**Methods:**

We performed a search in Pubmed and Cochrane on studies on reliability and validity of triage systems in children

**Results:**

The Manchester Triage System (MTS), the Emergency Severity Index (ESI), the Paediatric Canadian Triage and Acuity Score (paedCTAS) and the Australasian Triage Scale (ATS) are common used triage systems and contain specific parts for children. The reliability of the MTS is good and reliability of the ESI is moderate to good. Reliability of the paedCTAS is moderate and is poor to moderate for the ATS.

The internal validity is moderate for the MTS and confirmed for the CTAS, but not studied for the most recent version of the ESI, which contains specific fever criteria for children.

**Conclusion:**

The MTS and paedCTAS both seem valid to triage children in paediatric emergency care. Reliability of the MTS is good, moderate to good for the ESI and moderate for the paedCTAS. More studies are necessary to evaluate if one triage system is superior over other systems when applied in emergency care.

## Background

Large numbers of patients visit the emergency department. Consulting patients in the order of attending will, in a crowded emergency department (ED), lead to long waiting times for seriously ill patients. It is important to prioritise patients who are seriously ill and would be at increased risk of morbidity or even mortality due to delay in the initiation of treatment.

The aim of triage is to determine and classify the clinical priority of patients visiting the ED. [1] During a short assessment the nurse will identify signs and symptoms that determine the patient's urgency. The physician will see the patients in order of their urgency level. Patients requiring immediate care are identified. Moreover, patients are identified who can safely wait longer or who can be seen by another caregiver such as the general practitioner or nurse practitioner.

Triage systems are developed by expert opinion. [2-5], the lowest level of evidence, and are mainly based on the adult population visiting the ED. The Paediatric Canadian Triage and Acuity Scale (PaedCTAS) was especially modified for the paediatric population. [3] Several studies have investigated the reliability and validity of triage systems in children. [6-17]

The aim of this review is to provide an overview of the current scientific knowledge of triage systems for the broad population of children visiting the ED.

## Methods

We performed a search for literature in May 2009 using Cochrane and the following MeSH terms in Pubmed, "triage" [MeSH Terms] AND "emergency medical services" [MeSH Terms] AND ("infant" [MeSH Terms] OR "child" [MeSH Terms] OR "adolescent" [MeSH Terms]) AND (validity [All Fields] OR accuracy [All Fields]). Secondly we performed a wider search for "triage" [MeSH Terms] AND system [All Fields] AND "emergency medical services" [MeSH Terms] AND ("infant" [MeSH Terms] OR "child" [MeSH Terms] OR "adolescent" [MeSH Terms])

Studies were selected if they described a triage system for the broad population visiting the emergency care or reported about a study on reliability or validity of a triage system for emergency care, applied to the paediatric population. Studies on triage for a subpopulation were not included as well as for triage systems applied in the developing world. We included papers published between 1999 and 2009. Finally, reference lists of the included papers were checked for relevant publications using the same selection criteria.

## Results

The narrow search gave 44 hits, of which 12 were selected because of the title; one article was excluded following reading of the abstract. The broad search resulted in 112 hits of which six extra articles were selected.

### Triage systems in paediatric emergency care

Worldwide, the Manchester Triage System (MTS) [1,5,18], the Emergency Severity Index (ESI) [19,20] the Canadian triage and acuity scale (CTAS) [3] and the Australasian triage scale (ATS) [2] are consensus based and commonly used triage systems in emergency care. Although different criteria per triage system are used, they all sort patients into five urgency categories.

#### Manchester Triage system (MTS)

**Figure 1:**
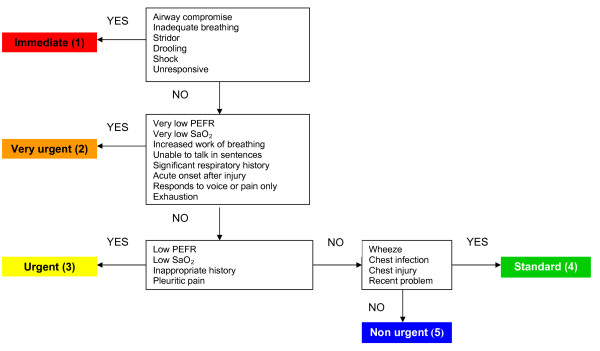
**Manchester Triage System flowchart Shortness of breath in children (Second edition)**. Reprinted with permission from Mackway-Jones K et al. Emergency Triage, Manchester Triage Group. Second edition. Oxford: Blackwell Publishing Ltd; 2006, p 134.[5]

The MTS contains 52 flowcharts presenting different presenting problems. Some flowcharts are specific for children, such as 'Worried parent', 'Abdominal pain in children', 'Crying baby', 'Shortness of breath in children', 'Limping child', 'Unwell child' and 'Irritable child'. The flowcharts contain general as well as specific discriminators, which are presenting signs or symptoms of the patient. General discriminators are life threat, pain, haemorrhage, conscious level, temperature and acuteness. [1] Specific discriminators are related to the presenting problems such as 'Increased work of breathing' (flowchart 'Shortness of breath in children') or 'Persistent vomiting' (flowchart 'Abdominal pain in children'). An example of a flowchart is provided in figure 1. (MTS flowchart 'Shortness of breath in children'). [5] The selected discriminator leads to an urgency level. Medical care should be delivered immediately for level 1, within 10 minutes for level 2, within 60 minutes for level 3, within 120 minutes for level 4 and within 240 minutes for level 5.

A second version of the MTS was published by the Manchester Triage group in 2006. [5] Some discriminators were modified or added (for example 'pain' in level 4 was modified to 'recent pain' for flowcharts in which pain is one of the discriminators). [5]

In a large validation study we identified subgroups of patients in which the validity of the MTS for children was low, such as young patients, patients with a non-traumatic presenting problem and older patients with fever. [16]

#### Emergency Severity Index (ESI)

The ESI is a 5-level triage system, developed in the United States. Level 1 stands for the highest acuity level and level 5 for the lowest acuity. Patients requiring immediate life-saving interventions are allocated into level 1 and must be seen immediately. Patients in a high risk situation, who are confused, lethargic, disoriented, have severe pain or distress or have deviated vital signs/PO_2 _are attributed to level 2. A physician should see these patients within ten minutes. Level 3 is for patients who are expected to require two or more resources. Level 4 is attributed if one resource is expected to be required and 5 if no resources are expected to be required. Resources can be diagnostics (for example lab tests, ECG, X-rays, CT scan etc), treatment (for example IV fluids, laceration repair) or specialty consultation. Patients triaged as level 3–5 can safely wait for several hours. [4]

In the fourth version of the ESI, a specific flowchart for children with fever was added. It uses age, the height of fever, the cause of fever and whether the child is immunized to determine urgency. Children younger than 28 days with a temperature >38.0°C are allocated to level 2. Children with fever aged 28 days – 3 months are assigned to level 2 or 3, depending on the hospital's institutional protocol. Children aged 3–36 months who are under immunized or who have no obvious source of fever and a temperature >39.0°C are allocated to level 3. [4]

#### Canadian Triage and Acuity Scale (CTAS)

In 2001 a specific guideline to triage children was added to the CTAS, (paedCTAS). Per presenting problem, specific criteria are provided to allocate patients to different urgency levels. For example for children presenting with respiratory distress, for level 1 signs are: inability to speak, cyanosis, lethargy or confusion, tachycardia or bradycardia, and hypoxemia with O_2 _saturation <90%. For level 2 the signs audible stridor, intermittent respiratory distress and audible wheezing, tachypnea, or cough are listed in order to select patients with respectively upper respiratory distress, congenital vascular anomalies and foreign bodies or lower airway concerns. Level 3 is for patients with moderate respiratory distress such as patients with pneumonia, bronchiolitis or croup. Level 4 and 5 do not contain criteria for patients with respiratory distress.

Medical care should be delivered immediately for level 1, within 15 minutes for level 2, within 30 minutes for level 3, within 60 minutes for level 4 and within 120 minutes for level 5. [21] A detailed recent description of the paedCTAS can be found at the website 

#### Australasian triage scale (ATS)

Formerly known as the National Triage Scale, the ATS provides criteria per urgency level. Most criteria are general but three criteria are specific for children: shocked child/infant should be allocated to level 1, all 'stable neonates' are allocated to level 3 as well as 'children at risk'. [22]

### Pain in triage

In the MTS as well as the paedCTAS pain plays an important role in urgency classification. Both systems allocate patients with severe pain to a level 2 urgency. Patients with moderate pain and patients with mild/acute pain (paedCTAS) or recent pain (MTS) are triaged into level 4. [3,5] The ESI allocates patients with severe pain to level 2. A lower pain score does not influence the ESI urgency level. [4] The Manchester pain scale correlated well with the Oucher pain scale, which is a common used and validated pain scale in emergency care. [23]

### Referral of low urgency patients to other caregivers

Besides prioritising urgent patients, triage systems are used to identify patients with a low urgency. These patients can safely wait, do not need urgent care and could as well be seen by another health professional. One study showed that the CTAS, when applied to adults and children is not valid to safely identify low urgency patients with the aim to refer them to other caregivers. [24] For other triage systems such as the MTS and the ESI, this question still needs to be answered.

### Research on reliability and validity of triage systems

Validity of a triage systems is determined by reliability (inter-rater agreement and intra-rater agreement) and whether or not the triage system can predict the true urgency (internal validity) The external validity determines the value of the system in different settings. [25] The inter-rater agreement is determined by the agreement in triage urgency level if multiple nurses triage one patient or patient scenario. The intra-rater agreement presents the agreement in triage urgency level if one triage nurse triages one case scenario at different points in time. The inter- and intra-rater agreement is dependent on the uniformity and completeness of a triage system and on how the triage nurse applies the system. Good training and instruction of the triage nurses can optimise the usage and interpretation of triage systems.

Inter- and intra-rater agreement are usually analysed using Cohen's kappa. Kappa provides a measure of agreement between observers, corrected for agreement expected by chance. [26] In case of an ordinal scale, which is the case when 5-level triage systems are studied, quadratic and linear weighted kappa analysis provide different weights per amount of disagreement. [27] If the inter-rater agreement between multiple observers is studied, the intraclass correlation coefficient (ICC) can be used. It can easily be calculated using SPSS and is equivalent to a quadratic weighted kappa, under certain conditions. [28]

To assess validity, a 'gold standard' as a proxy for urgency has to be defined. Since it is difficult to determine the 'true urgency', different approaches are currently used to assess validity. Outcome measures such as hospitalisation, ICU admission, resource uses, total length of stay at the ED or costs of an ED consultation are used. [6,8,13]

We studied the validity of the MTS in children in a large prospective observational study by comparing the MTS urgency level with a predefined, independently assessed reference standard for urgency. [16] We defined the highest urgency level for patients with deviated vital signs according to the PRISM (Paediatric Risk of Mortality), [29] patients with a potentially life threatening diagnosis were defined as level 2, patients were allocated to level 3 or 4 depending on if they were hospitalised after ED consultation and the amount of diagnostics and therapeutic interventions performed at the ED. Patients allocated to level 5 did not meet the criteria for level 1 or 2, were not hospitalised, and no diagnostics or therapeutic interventions were performed during their ED visit. A detailed description of the reference standard was published before. [16] It is important to triage a patient and to assess the reference independently, in order not to overestimate validity. [25]

Assessing urgency per case by experts is another way to assess validity. However, these judgements are quite dependent on the used protocols in the hospital and the personal experience of the expert.

Validity can be expressed in sensitivity and specificity of a triage system. Sensitivity presents the ability for a triage system to identify high urgent patients. Specificity presents the ability for a triage system to identify patients with low urgent problems. The 'Likelihood Ratio for a positive test results' (LR+) represents the ratio between the chance on a high urgency test result in patients with a true high urgency and the chance of a high urgency test results in patients with a true low urgency. [25,30]

Validity is analysed in some studies by assessing agreement between the triage system urgency and a reference urgency, using kappa statistics. [6,13] Van der Wulp et al suggested a triage weighted kappa in which under-triage (when the triage urgency is lower than the reference urgency) is weighted as more severe than over-triage (when the triage urgency is higher than the reference standard urgency). [31] Lee at al proposed a weighted scheme (error weights) for a 3-level triage system, in which under-triage was weighted twice as over-triage. They calculated sensitivity, specificity, positive and negative predictive value incorporating these error weights. [32]

### Reliability and validity of triage systems in paediatric emergency care

Table 1 and 2 provide an overview of studies on reliability and validity of triage systems when applied to children.

**Table 1:** Studies on reliability of the ESI, CTAS, MTS and ATS in paediatric emergency care

Country	N scenarios, raters (response rate)*	Triage system/population	Study design	Results ^‡^
Australia [34]	14 scenarios, 178 nurses**	ATS, children	7 paper, 7 computer based scenarios	K 0.40 (paper) K 0.58 (computer)
Australia [35]	8 scenarios, 97 nurses (44%)	ATS, children	Written case scenarios	K 0.21
USA [6]	20 scenarios^†^	ESI version 3, children	Written case scenarios	K_w _0.84–1.00
USA [6]	272 patients	ESI version 3, children	Simultaneous triage	K_w _0.59 (95% CI 0.55–0.63)
Canada [9]	54 scenarios, 18 nurses (62%)	PaedCTAS children	Written case scenarios	K_w _0.51 (95% CI 0.50–0.52)
Canada [10]	499 patients	PaedCTAS children	Simultaneous triage	Lineair K_w _0.55 (95% CI 0.48–0.61) Quadratic K_w _0.61 (95% CI 0.42–0.80)
The Netherlands [15]	50 scenarios, 48 nurses (87%)	MTS adults and children	Written case scenarios	K_w _0.62
The Netherlands [17]	20 scenarios, 43 nurses (100%) 198 patients	MTS in children	Written case scenarios Simultaneous triage	Quadratic K_w _0.83 (95% CI 0.74–0.91) Quadratic K_w _0.65 (95% CI 0.56–0.72)

* For studies using the written case scenario method

** Compliance rate not described in paper † N raters and compliance rate not described in paper

‡ K kappa, K_w _Weighted kappa,

ATS = Australasian Triage Scale, ESI = Emergency Severity Index, MTS = Manchester Triage System, PaedCTAS = Paediatric Canadian Triage and Acuity Scale

Kappa/weighted kappa: poor if K ≤ 0.20, Fair if 0.21 ≤ K ≤ 0.40, moderate if 0.41 ≤ K ≤ 0.60, good if 0.61 ≤ K ≤ 0.80 very good if K>0.80. (95% confidence interval)

**Table 2:** Studies on validity of the ESI, CTAS, MTS in paediatric emergency care

Country	N, patients	Triage system	Design	Outcome measure	Conclusion
Canada [8]	807/560	PaedCTAS	Before and after design, prospective study	Admission rate, medical interventions, and PRISA score, comparison with previous used triage tool (4 level)	Previous triage tool had better ability to predict admission than paediatric CTAS
Canada [11]	58,529	PaedCTAS	Retrospective	Admission, ICU admission Length of stay (LOS)	Good correlation between urgency and admission, ICU admission and LOS
Canada [33]	1,618	PaedCTAS	Retrospective	Costs of resource utilization	PaedCTAS urgency level correlates well with resource utilization
USA [6]	510	ESI (version 3) Children	Prospective triage, retrospective chart review	Admission rate, medical interventions, PRISA score, comparison with used triage tool	ESI score predicts resource use, length of stay, and admission to hospital
The Netherlands [14]	1,065	MTS	Retrospective	Reference standard for urgency *	Sensitivity 63% Specificity 78%
The Netherlands [16]	17,600	MTS	Prospective	Reference standard for urgency *	Sensitivity 63% Specificity 79%

ESI = Emergency Severity Index, MTS = Manchester Triage System, PaedCTAS = Paediatric Canadian Triage and Acuity Scale

* Reference standard based on vital signs, diagnosis, resource use, admission rate, and follow-up

The ESI has a moderate (actual simultaneous triage) to good (written case scenarios) reliability when applied to triage children. ESI urgency levels are correlated to resource use, length of stay at the ED. [6] The paedCTAS has a moderate inter-rater agreement using actual simultaneous triage. [9,10]

Several validity studies of triage systems in children show a correlation of urgency levels with admission. A large study on the validity of the paedCTAS showed that 90% of the patients admitted to the PICU, were triaged as urgency level 1 or 2.

3 patients out of the total 58,529 were 'incorrectly' triaged as level 4 or 5. [11] Patients triaged as level 3–5 were admitted in 6% (out of 400 patients) using the ESI. [6], and in 7% (out of 510 patients) and 6% (out of 53,846 patients) using the paedCTAS. [8,11]

Patients triaged as level 1 or 2 were admitted in 36% (out of 110 patients) using the ESI [6], and in 30% (out of 27 patients). [8] and 41% (out of 4683 patients) using the paedCTAS. [11] Percentage admission per urgency level is comparable between triage systems.

Furthermore, paedCTAS urgency levels are related to resource use and length of stay, although length of stay was shorter for level 1 patients compared to level 2 patients (191 minutes versus 250 minutes). [11,33] The ATS showed a poor to moderate reliability. [34,35] We did not find studies on the validity of the ATS for children.

The inter-rater agreement of the MTS in adults and children was studied in the Netherlands and showed a good to excellent reliability. [15,17] For children the inter-rater agreement of the MTS is good (simultaneous triage of actual patients) to excellent (written case scenarios). Validity, expressed in agreement between the MTS and reference standard for urgency, shows 34% correct triage, 54% were over-triaged and 12% under-triaged. Sensitivity was 63% (95% CI 59–66) and specificity 79% (95% CI 79–80). [16]

## Discussion

Several triage systems are extensively used to triage children at the emergency department. Several studies are performed to assess the reliability and validity of these systems in children.

The aim of triage is to identify high urgent patients. Triage systems that show a large proportion of under-triage or perform a low sensitivity (real high urgent patients are triaged as low urgent) are therefore unsafe.

Since it will be difficult for a triage system to reach 100% sensitivity and specificity, a good balance between over- and under-triage is important. A high sensitivity may result in a low specificity resulting in many patients with real low urgent problems who will be treated as high urgent. This may result in long waiting times for real high urgent patients.

Since outcome measures used for validity studies are different, a comparison between triage systems cannot be made on how they predict 'true' urgency. However, from the available studies and the design of the triage systems, some points can be made. The ESI performs a moderate to good inter-rater agreement. [6] Inter-rater agreement for the paedCTAS is moderate when written case scenarios are used. When the paedCTAS is studied using real life scenarios, results are similar to the inter-rater agreement of the ESI. Reliability is good for the MTS [15,17] and poor to moderate for the ATS.(Table 1)

Validity is confirmed for the MTS and paedCTAS. Validity of the paediatric fever criteria of the ESI was not studied. Since patients presenting with fever are 15% of the paediatric population [16], it is important to study these fever criteria as well. (Table 2) The MTS is both detailed and objective and discriminators are organized in flowcharts of presenting problems. The system contains several specific flowcharts for children. [5].

### Methodology

From a methodological view triage can be seen as a diagnostic test; predicting 'true' urgency. In that way sensitivity and specificity must be used as measures of performance. [30] A disadvantage of this method is that urgency levels following from a 5 level triage system should be dichotomised. When one chooses to combine the two highest levels of a triage system as 'high urgency' and the three lowest as 'low urgency', a distinction between the two highest levels and between the three lowest levels is not made anymore. However, the aim of triage is to identify true high urgent patients. A misclassification in the two highest urgency levels (level 1 or level 2) is clinically less important than a misclassification from level 2 to level 3, 4 or even 5. By dichotomising the 5 urgency levels and calculating sensitivity and specificity, weights are incorporated. Moreover sensitivity and specificity are very commonly used in diagnostic research and therefore easily interpretable by most users. [30]

### Implementation

Implementation includes application of the system to all patients and compliance to the advice for urgency by the ED nurses. The implementation of the triage system in practise is important for the triage process. Patients who enter the emergency department should be triaged as soon as possible. If children are sitting in a waiting room without being triaged, potentially dangerous delay in treatment can occur for potentially serious diseases.

Especially in a crowded emergency department it is important that there is a triage nurse whose primarily role is triage. She will perform a rapid assessment (30–60 seconds) and long conversations with patients should be avoided. [5] The founders of the ESI and the MTS claim that a complete assessment does not need to be done at the initial triage station, although sufficient information should be gained to be able to determine the correct triage category. [4,5] Vital signs should be completed on all paediatric patients at some time during their emergency visit. [3] The triage nurse will take care that that all patients entering are directly triaged (within 10 minutes of arrival) [3] while other nurses take care of further observation and treatment of patients.

As for implementation of clinical prediction rules, certain criteria should be met for successful implementation. At first predictions of the triage system should be better than that of the users. Secondly, users should feel that the system is valid (face validity). Since wide validation of triage system is often lacking, this is a point for improvement. Thirdly the system should be user friendly. The best predictors of a rule to be used in practice are the familiarity acquired during training, the confidence in the usefulness of the rule, and the user-friendliness of the rule. [36,37]

Computerized triage showed a better agreement in correct triage outcome, compared to triage without the support of a computerized application. [38] Application of the paedCTAS using a computerized application (Staturg) resulted in a better reliability of the system. [9] Therefore, a computerized application of a triage system should be used. [39] Especially the MTS and the CTAS are complex systems for which several questions should be answered before a triage advice is suggested.

## Conclusion

Several systems are available for triage in paediatric emergency care. The MTS, ESI and CTAS contain parts specific for children. Evaluation of a triage system concerns research of reliability and validity. The MTS and paedCTAS both seem valid to triage children in paediatric emergency care. Available studies show that reliability of the MTS is good, is moderate to good for the ESI, moderate for the paedCTAS and poor to moderate for the ATS. More research is needed on the reliability and validity of triage systems when applied to children especially if they are used to identify low urgent patient for referral to another caregiver.

## Abbreviations

MTS: Manchester Triage System; ESI: Emergency Severity Index; PaedCTAS: Paediatric Canadian Triage and Acuity Scale; ATS: Australasian Triage Scale; ED: Emergency Department.

## Competing interests

The authors declare that they have no competing interests.

## Authors' contributions

MV and HM designed the review; MV drafted the paper and performed the literature search, HM revising it critically for important intellectual content. All authors read and approved the final manuscript.
